# Understanding the Contribution of Zinc Transporters in the Function of the Early Secretory Pathway

**DOI:** 10.3390/ijms18102179

**Published:** 2017-10-19

**Authors:** Taiho Kambe, Mayu Matsunaga, Taka-aki Takeda

**Affiliations:** Division of Integrated Life Science, Graduate School of Biostudies, Kyoto University, Kyoto 606-8502, Japan; matsunaga.mayu.76x@st.kyoto-u.ac.jp (M.M.); takeda.takaaki.77s@st.kyoto-u.ac.jp (T.T.)

**Keywords:** ZNT/Solute carrier family 30 member (SLC30A), ZIP/SLC39A, early secretory pathway, ER stress, unfolded protein response (UPR), zinc-requiring ectoenzymes, tissue non-specific alkaline phosphatase (TNAP), metallation

## Abstract

More than one-third of newly synthesized proteins are targeted to the early secretory pathway, which is comprised of the endoplasmic reticulum (ER), Golgi apparatus, and other intermediate compartments. The early secretory pathway plays a key role in controlling the folding, assembly, maturation, modification, trafficking, and degradation of such proteins. A considerable proportion of the secretome requires zinc as an essential factor for its structural and catalytic functions, and recent findings reveal that zinc plays a pivotal role in the function of the early secretory pathway. Hence, a disruption of zinc homeostasis and metabolism involving the early secretory pathway will lead to pathway dysregulation, resulting in various defects, including an exacerbation of homeostatic ER stress. The accumulated evidence indicates that specific members of the family of Zn transporters (ZNTs) and Zrt- and Irt-like proteins (ZIPs), which operate in the early secretory pathway, play indispensable roles in maintaining zinc homeostasis by regulating the influx and efflux of zinc. In this review, the biological functions of these transporters are discussed, focusing on recent aspects of their roles. In particular, we discuss in depth how specific ZNT transporters are employed in the activation of zinc-requiring ectoenzymes. The means by which early secretory pathway functions are controlled by zinc, mediated by specific ZNT and ZIP transporters, are also subjects of this review.

## 1. Introduction

Zinc is an essential trace element that is required for a large variety of cellular processes [1,2]. Approximately 10% of the eukaryotic proteome requires zinc for cellular activity [3,4], and thus any disturbance in zinc homeostasis can result in disease, including cancer, neuronal degeneration, chronic inflammation, hypertension, osteoarthritis, and age-related macular degeneration. A diverse range of symptoms is also found in cases of zinc deficiency [1,2,5,6,7,8]. The biological functions of zinc can be grouped into three major categories, structural, catalytic, and regulatory. However, the molecular basis of how zinc engages in such diverse functions is still far from being completely understood [1,2].

At the cellular level, zinc plays a pivotal role in the function of a variety of subcellular compartments, one of which is the early secretory pathway constituted by the endoplasmic reticulum (ER), the Golgi apparatus, and other intermediate organelles, such as the ER-Golgi intermediate compartment. Zinc homeostasis in the lumen of these compartments requires a transport system to translocate zinc across biological membranes. In vertebrates, Zn transporters (ZNTs)/Solute carrier family 30 member (SLC30A) and Zrt- and Irt-like proteins (ZIPs)/SLC39A are widely recognized as being critical transporters in zinc metabolism under physiological conditions [1,2,9]. Both of these proteins are clearly important for zinc metabolism involved in early secretory pathways. This review outlines the functions of ZNT and ZIP transporters in the regulation and function of secretory pathways, in particular, the early secretory pathway, focusing on several recent aspects of the molecular processes underlying the ER stress response, as well as the activation of zinc-requiring ectoenzymes. Zinc transporters also play important roles in secretory granules/vesicles that contain high amounts of zinc, such as insulin granules, synaptic vesicles, and secretory vesicles involved in milk secretion; these are discussed in this review for comparison. Further details of these transporters can be found in other comprehensive reviews of zinc transporters [1,10,11,12].

## 2. Brief Overview of the Properties of ZNT and ZIP Transporters

**Figure 1:**
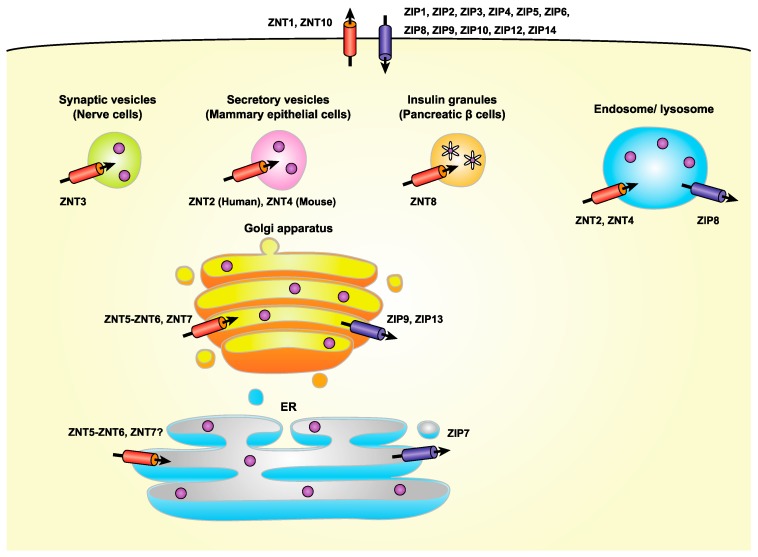
Subcellular localization of ZNT and ZIP transporters. ZNT transporters move cytosolic zinc into the lumen of vesicles involved in the early secretory pathway, including the endoplasmic reticulum (ER), Golgi apparatus, as well as into cytoplasmic vesicles/granules such as synaptic and secretory vesicles and insulin granules, in which specific ZNT proteins are localized. ZNT5 and ZNT6 form heterodimers to transport zinc. ZIP transporters move zinc in the opposite direction. In contrast to the specific localization of ZIP9, ZIP13, and ZIP7 in the Golgi apparatus and the ER, the subcellular location of ZNT5-ZNT6 heterodimers has not been definitively determined.

In mammals, there are nine ZNT and 14 ZIP transporters that play distinct roles in the maintenance of systemic, cellular, and subcellular zinc homeostasis. These transporters act in a cell or tissue-specific manner, and are developmentally regulated [1,2,9] (Figure 1). ZNTs transport zinc from the cytosol into either the lumen of intracellular compartments or the extracellular milieu, whereas ZIPs transport zinc in the opposite direction. Zinc transport by ZNT and ZIP transporters is coordinately controlled through precisely timed increases or decreases in their expression, and by their precise subcellular localization [2,13]. A growing body of evidence has shown that cooperative zinc transport across biological membranes mediated by both transporters contributes to the control of expression, localization, and functional activity of target proteins [1]. The molecular features of ZNT and ZIP transporters have been extensively summarized in other review papers [1,2], and thus only their main features are outlined briefly here.

**Figure 2:**
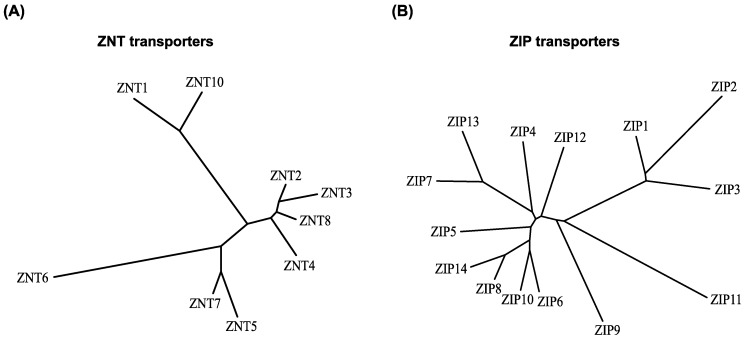
Phylogeny of ZNT and ZIP transporters. The neighbor-joining phylogenetic tree was constructed using ClustalW (http://clustalw.ddbj.nig.ac.jp/index.php?lang=en) protein alignment. (**A**) ZNT and (**B**) ZIP transporters. Subfamilies and subgroups are designated according to the text.

Based on the three-dimensional structure of the *Escherichia coli* homolog YiiP, ZNT transporters are predicted to have six transmembrane (TM) helices (TM helices I-VI) [14,15,16,17,18]. ZNT transporters function as zinc/proton exchangers [19,20], and can form homodimers or heterodimers [21,22,23,24]. With respect to the zinc transport mechanism used by YiiP, two models have been proposed, the alternative access mechanism model, in which the TM helices form inward- and outward-facing conformations [17,18], and the allosteric mechanism model, in which cytosolic zinc binding induces a scissor-like movement of the homodimers and interlocks the TM helices at the dimer interface [15,16]. ZNT transporters likely transport zinc using either of the two proposed mechanisms. As has been observed for YiiP, which has an intramembranous zinc-binding site formed by TM helices II and V, ZNT transporters are also thought to have a conserved intramembranous zinc-binding site, which is indispensable for zinc transport activity [19,20,25,26]. The intramembranous zinc-binding site in most ZNT transporters consists of two His and two Asp residues in TM helices II and V [19,27]. Interestingly, ZNT10 has an Asn residue in TM helix II instead of His, which confers the ability to transport manganese [28], as has also been seen for the homologous bacterial protein [29]. The nine ZNT transporters belong to the cation diffusion facilitator (CDF) family of transporters, which are classified into three subgroups, namely Zn-CDF, Zn/Fe-CDF, and Mn-CDF [14,30]. All of the ZNT transporters are classified as being Zn-CDF members (although ZNT10 is a manganese transporter), and, based on their sequence similarities, can be further subdivided into four groups: (i) ZNT1 and ZNT10, (ii) ZNT2, ZNT3, ZNT4, and ZNT8, (iii) ZNT5 and ZNT7, and (iv) ZNT6, [14,31,32] (Figure 2). Of interest to this review, some characteristics of the transporters, such as subcellular localization, are conserved in the members of the same group [32] (Figure 1).

Computational analysis suggested that ZIP transporters have eight TM helices [33], and this was confirmed by the first three-dimensional structure reported for a ZIP transporter homolog in bacteria (*Bordetella bronchiseptica*) [34]. The structure shows that the ZIP transporter has a novel 3 + 2 + 3 TM architecture with a binuclear metal center, in which two His residues, one each in TM helices IV and V, form two intramembranous zinc binding sites [34]. As in ZNT transporters, ZIP transporters form functional homo- or heterodimeric complexes, which are essential for their zinc transport ability, although no dimer formation was seen in the crystal structure [34,35,36]. ZIP transporters may function as selective electrodiffusion channels [37], or as zinc/bicarbonate symport transporters [38,39,40]. However, their definitive mode of transport has not yet been completely elucidated. Phylogenetic analysis classifies the fourteen ZIP transporter members into four subfamilies, namely ZIP I (ZIP9), ZIP II (ZIP1-ZIP3), LIV-1 (ZIP4-ZIP8, ZIP10, ZIP12-ZIP14), and gufA (ZIP11) [1,41]. In the LIV-1 subfamily, features of their extracellular domains further classify the proteins into four subgroups as follows: (i) ZIP4 and ZIP12, (ii) ZIP8 and ZIP14, (iii) ZIP5, ZIP6, and ZIP10, and (iv) ZIP7 and ZIP13 [42] (Figure 2). Members of the LIV-1 subfamily have an extended extracellular N-terminus, whose structure has been solved only in the case of ZIP4 [42]. The extracellular portion of ZIP4 can form homodimers without the need for TM helices [42], which may facilitate dimerization. Interestingly, in ZIP transporters belonging to subgroup (iii), a prion-like domain is present in the extracellular N-terminal portion proximal to the first TM helix; hence, there is an evolutionary link between these transporters and the prion protein [43]. ZIP8 and ZIP14 in subgroup (ii) have the ability to transport manganese [39,40], because they have a Glu residue in TM helix V rather than a His residue [1,44]. The His residue is therefore involved in metal substrate specificity, because of its contribution to forming intramembranous zinc binding sites [34].

Over the last two decades, the physiological roles of ZNT and ZIP transporters and their involvement in disease pathology have been clarified at the molecular level, as has been described elsewhere [1,2,8,9], and a deeper understanding will likely come in the future.

## 3. Regulation of Zinc Homeostasis by Zinc Transporters in the Early Secretory Pathway

Approximately one-third of all the cellular proteins in eukaryotes are targeted to the ER, and thus the early secretory pathway [45], in which nascent proteins are folded, assembled, and modified during their trafficking to final destinations. Importantly, a considerable proportion of the secretome requires zinc as a structural and catalytic cofactor. Moreover, resident chaperones require zinc for modulation and potentiation of their functions [46,47,48]. Hence, any disruption of zinc homeostasis in the early secretory pathway can cause and exacerbate ER stress [49,50], and trigger the unfolded protein response (UPR) in cells. Therefore, elaborate regulatory mechanisms are used to control zinc homeostasis in the early secretory pathway. Accumulating evidence clearly shows that both ZNT and ZIP transporters play crucial roles in this process [49,50,51,52,53], and this is summarized in this section.

**Figure 3:**
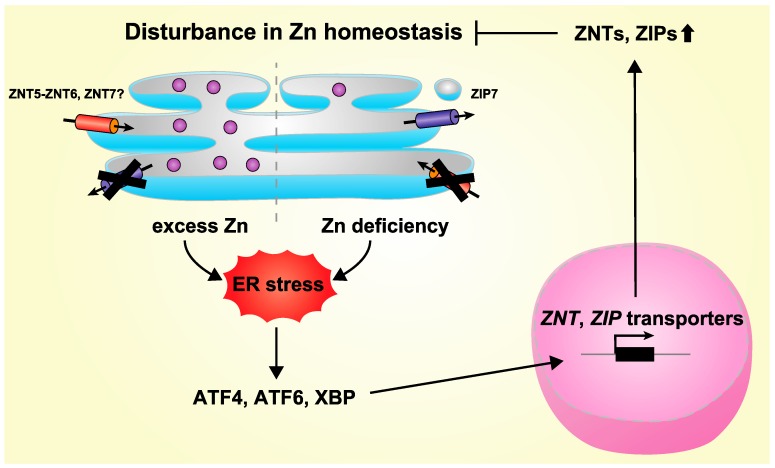
Model of feedback regulation for the maintenance of zinc homeostasis in the ER (in the early secretory pathway). A disturbance in zinc homeostasis, such as zinc deficiency or zinc overload, in the ER (and perhaps in the early secretory pathway) induces homeostatic ER stress. The unfolded protein response (UPR) leads to the activation of transcription factors such as ATF4, ATF6, and XBP1, and increases the transcription of several ZNT and ZIP transporter genes. These activities of ZNT and ZIP transporters then contribute to the maintenance of zinc homeostasis in the ER (and in the early secretory pathway), and thus attenuate homeostatic ER stress. Zn: zinc.

With the exception of ZNT1 and ZNT10 (which are members of ZNT subgroup (i) described above [28,54]), ZNT transporters are mainly localized to intracellular compartments. Of these, ZNT5, ZNT6, and ZNT7 (members of ZNT subgroups (iii), and (iv) described above) are involved in the early secretory pathway [12,55]. ZNT5 has been shown to be mainly localized to coat protein complex II (COPII) vesicles and the Golgi apparatus [56], whereas ZNT6 is localized to the Golgi apparatus [57], although these ZNTs can also form heterodimers as functional complexes [21,22] (Figure 1). The actual subcellular localization of these heterodimers has however been poorly investigated. ZNT7 is also located in the Golgi apparatus [58], and a recent study indicates that it is also localized to the sarco(endo)plasmic reticulum (S(E)R) [52] (Figure 1). These ZNT transporters are employed as zinc entry routes in the early secretory pathway, suggesting that a lack of them would be potentially to elicit an ER stress response. In fact, it has been clearly shown that cells lacking these ZNT transporters do exhibit an exacerbated ER stress responses [50,59] (Figure 3). A similar exacerbation of ER stress is found in yeast lacking ZNT orthologs [49,60], which highlights the fact that the important role these ZNT transporters play in maintaining zinc homeostasis in the secretory pathway is well-conserved among subgroups. However, there remains the interesting questions of how and where these ZNT transporters transport zinc, and their association with ER stress, because ZNT5, ZNT6, and ZNT7 all appear to be principally localized to the Golgi apparatus [57,58,61]. Recent studies have shown that another ZNT transporter, either ZNT3 or ZNT10, may play a protective role in ER stress-induced toxicities [62,63], although their contributions to the early secretory pathway have not yet been clarified.

In contrast to ZNT transporters, most of which are located in intracellular compartments, most, but not all, ZIP transporters are found on the plasma membrane. Of the 14 ZIP transporters, ZIP7, ZIP9, and ZIP13 (the ZIPI subfamily and the LIV-1 subgroup (iii) described above) are involved in the early secretory pathway [64,65,66,67] (Figure 1), although recent reports also indicate that ZIP9 is also found localized on the plasma membrane where it serves as a membrane androgen receptor [68] and that ZIP13 can also be found in intracellular vesicles [69]. It has also been suggested that ZIP11 can localize to the Golgi apparatus [70], but this has not yet been thoroughly established. Accordingly, this protein is not further discussed here. ZIP7, ZIP9, and ZIP13 are known to release zinc, which is stored in the early secretory pathway, into the cytosol in response to various stimuli, thus contributing to the signaling function of zinc [66,71,72,73]. Importantly, ZIP7, ZIP9, and ZIP13 are also thought to contribute to homeostatic maintenance of the secretory pathway. In this regard, ZIP7, which is located in the ER, plays an indispensable role in the proper regulation of ER function, through the fine-tuning of zinc homeostasis [51,53]. The loss of ZIP7 probably increases zinc levels in the ER, which triggers zinc-dependent aggregation of protein disulfide isomerase, leading to ER stress [53] (Figure 3). This critical functional role of ZIP7 in ER homeostasis contributes to self-renewal processes of intestinal epithelium [51] and appropriate epidermal development [53]. ZIP7 is also involved in the induction of ER stress by mediating the redistribution of zinc into the cytosol from the S(E)R in cardiomyocytes under hyperglycemic conditions [52]. Consistent with the involvement of ZIP7 in ER stress, the yeast ZIP7 homolog, yKE4, has been shown to be involved in ER stress responses [74]. Similarly, Catsup, a Drosophila ZIP7 ortholog, which plays a crucial role in catecholamine synthesis, is also involved in the ER stress response [75,76]. Along with ZIP7, Golgi-localized ZIP9 is also thought to contribute to secretory homeostasis [67], and likewise, ZIP13 contributes directly to zinc homeostasis in the early secretory pathway by mobilizing zinc from the Golgi apparatus, or indirectly by releasing zinc from intracellular vesicles [69]. Recently, although it is localized to the plasma membrane [77,78], ZIP14 has also been shown to play a significant role in the adaptation to ER stress [79,80], suggesting that zinc homeostasis in the early secretory pathway might be indirectly controlled by ZIP transporters that are located in other subcellular regions.

With respect to the direction of zinc transport mediated by ZNT and ZIP transporters, both decreases and increases in zinc levels in the early secretory pathway exacerbate its proper functioning and thus either increases or decreases in zinc levels will result in the homeostatic ER stress response. The molecular basis underlying this phenomenon may be explained by changes in the activity of chaperone proteins that are either positively or negatively regulated by zinc [46,47,48], although this has not yet been completely elucidated. 

## 4. Regulation of Expression of ZNT and ZIP Transporters by ER Stress

Based on the crucial functions of zinc mobilized by ZNT and ZIP transporters in the early secretory pathway, it is easy to imagine that the transcription of ZNT and ZIP genes would be regulated by homeostatic ER stress. In fact, *ZNT5* transcription increases in response to inducers of ER stress, and its promoter harbors a UPR element, which serves as the binding site for the transcription factor XBP-1 [50] (Figure 3). *ZIP14* transcription is also induced by inducers of ER stress [79,80], and its promoter also has several ER stress response elements, to which the transcription factors ATF6 and ATF4 bind [79,80]. Moreover, the treatment with inducers of ER stress or *N*,*N*,*N*′,*N*′-tetrakis(2-pyridylmethyl)ethylenediamine, a zinc chelator which also causes ER stress, has been shown to induce the expression of ZIP3, ZIP7, ZIP9, ZIP13, and ZIP14, as well as ZNT3, ZNT6, ZNT7, and ZNT10 [51,62,63,79,80], although the elements responsible for the induction have not yet been identified in the promoter regions of these genes. Several homologues of both types of transporter have been shown to be increased by inducers of ER stress [81]. Thus, the fine-tuning of zinc homeostasis by these zinc transporters in the early secretory pathway, as well as the regulation of their expression triggered by homeostatic ER stress, are important control mechanisms in maintaining homeostasis [12].

## 5. Importance of ZNT Transporters in the Activation of Ectoenzymes in the Early Secretory Pathway

Zinc-requiring ectoenzymes, which are defined here as secretory, membrane-bound, and organelle-resident enzymes, have attracted considerable attention because they play crucial roles in various physiological functions, and in a number of pathological processes, such as cancer progression, and metastasis [1,55]. Thus, they are regarded as potential therapeutic targets in the treatment of diseases [82,83,84,85,86]. Moreover, the activities of some zinc-requiring ectoenzymes, e.g., alkaline phosphatases (ALP), may be used as clinical markers to reflect systemic zinc status [87,88]. These enzymes are synthesized in the early secretory pathway, at which point they acquire zinc for their activity, before being trafficked to the plasma membrane via the constitutive secretory pathway [12,55]. How zinc is made available to zinc-requiring ectoenzymes is largely unknown, but the importance of ZNT transporters has been partially clarified in the activation of specific enzymes. This section addresses these specific ectoenzyme activation processes in detail.

### 5.1. ZNT Transporters Involved in Zinc-Requiring Ectoenzyme Activation

Zinc-requiring ectoenzymes likely become active by coordinating with zinc at their active site (i.e., they become metallated) during the secretory process. Zinc coordination is generally achieved by the interaction of zinc with three or four amino acids, including His, Asp, and Glu residues [4,89,90], which must undergo precise regulation for the conversion from the apo- to holo-forms. When compared with cytosolic zinc-requiring enzymes, zinc-requiring ectoenzymes require more complicated and elaborate regulatory processes involving zinc mobilization, because their activation process requires at least two types of zinc transporters that involve two biological membranes. In the first process, zinc transport from the extracellular milieu to the cytosol (i.e., ZIP transporters) occurs, and in the second process zinc transport from the cytosol to the lumen (i.e., ZNT transporters) occurs. Information regarding the identity of the ZIP transporters involved is lacking, while information relating to the ZNT transporters is accumulating. Three ZNT transporters, constituting two independent complexes, have been shown to be indispensable in ectoenzyme metallation; one complex is formed by ZNT5 and ZNT6 as a heterodimer, in which ZNT6 operates as an auxiliary subunit, and the other is formed by ZNT7 homodimers [21,91,92]. Both of these ZNT complexes can specifically activate several zinc-requiring enzymes, such as tissue-nonspecific ALP (TNAP) and placental ALP, as well as autotaxin (ATX) [24,56,93]. These three enzymes have similar active site geometry with a bimetallic core, which consists of two zinc ions, one of which is coordinated by one Asp and two His residues, and the other coordinated by one His and two Asp residues (Table 1), although ALPs and ATX catalyze different enzymatic reactions and have different biological roles. Based on these enzymes, it could be hypothesized that specific regulation mechanisms are operative in this bimetallic core enzyme family during conversion from the apo- to holo-enzymes. Both ZNT complexes, however, can activate other zinc-requiring ectoenzymes, such as matrix metalloproteinase (MMP)-9 and probably MMP-2 [24], and thus, they likely play a critical role in the activation of many zinc-requiring ectoenzymes in the early secretory pathway.

However, some enzymes can be metallated by zinc and become activated through different pathways involving these zinc transporters. For example, carbonic anhydrase IX (CAIX) can acquire zinc via ZNT4 homodimers, in addition to ZNT5-ZNT6 heterodimers and ZNT7 homodimers [24]. ZNT4 homodimers may also be involved in carbonic anhydrase VI maturation [94]. These findings are interesting for two reasons. The first is that ZNT4 homodimers have multifunctional roles depending on their subcellular localization. For example, ZNT4 was originally reported to be localized to late endosomes [95], where it has a role in reducing cytosolic zinc toxicity [25,96], but it has also been shown to be involved in secretory pathways involving the *trans*-Golgi network, cytosolic vesicles, and probably other secretory vesicles. In addition, it has been shown to be involved in zinc secretion into breast milk in mice [97,98]. The second is that ZNT4 homodimers can become functionally equivalent to ZNT5-ZNT6 heterodimers or ZNT7 homodimers in the activation of specific ectoenzymes, including CAIX, in the early secretory pathway. The involvement of ZNT4 in CAIX activation is specific because ZNT2 expression failed to result in CAIX activation [24].

### 5.2. Insight into the Activation of TNAP and Other Ectoenzymes by ZNT5-ZNT6 Heterodimers and ZNT7 Homodimers

What affects the metallation of TNAP via ZNT5-ZNT6 heterodimers or ZNT7 homodimers? This question remains to be fully resolved but some important insights have been made to date. First, the number of zinc ions and their coordination manner at the active site does not seem to affect the activation process mediated via ZNT5-ZNT6 heterodimers or ZNT7 homodimers. The ALP protein possesses two zinc ions at the active site (zinc bimetallic core), which are coordinated by His, His, and Asp residues or Asp, Asp, and His residues, as described above [55], whereas MMP-9 has a single zinc ion at the active site, which is coordinated by three His residues (MMP-9 has another zinc ion that acts as a structural component) [55] (Table 1). CAIX, which can be activated by ZNT4 homodimers, ZNT5-ZNT6 heterodimers and ZNT7 homodimers [24], as described above, possesses a single zinc ion coordinated by three His residues at the active site, supporting this notion.

**Figure 4:**
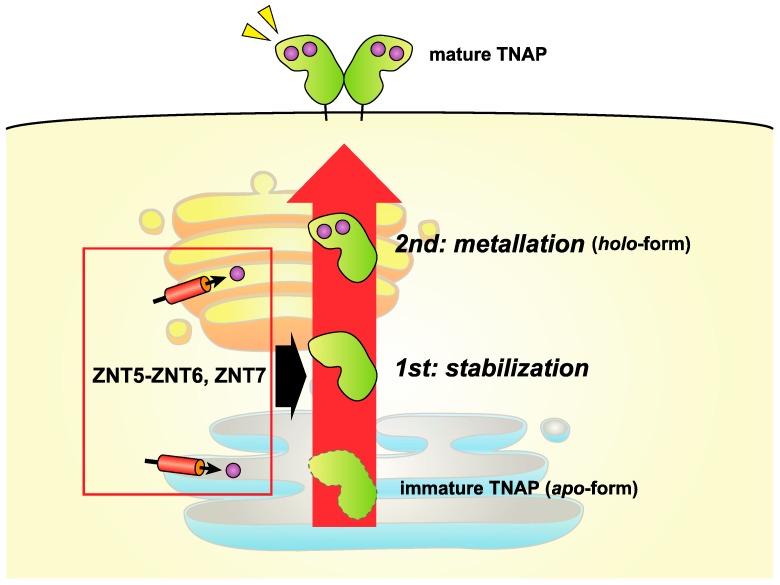
ZNT5-ZNT6 heterodimers and ZNT7 homodimers function to activate tissue-nonspecific ALP (TNAP) in a two-step mechanism. TNAP is specifically activated in a two-step mechanism involving ZNT5-ZNT6 heterodimers and ZNT7 homodimers as follows: first, the apo-form of TNAP is stabilized by either ZNT5-ZNT6 heterodimers or ZNT7 homodimers; second, the apo-form of TNAP is converted to the *holo*-form by zinc metallation. The PP-motifs in ZNT5 and ZNT7 likely play important roles in this process (see text). TNAP possesses a bimetallic core, is dimeric, and is localized to the plasma membrane via a glycophosphatidylinositol anchor. The subcellular localizations of ZNT5-ZNT6 heterodimers or ZNT7 homodimers have not been well defined. Zn: zinc.

Second, a specific motif may be significantly involved in TNAP activation via ZNT5-ZNT6 heterodimers and ZNT7 homodimers [55,99]. In cells lacking both ZNT complexes, the TNAP protein is destabilized, although it is not destabilized by zinc deficiency [93]. These data show that both ZNT complexes can stabilize the TNAP protein, in addition to supplying it with zinc. In other words, the TNAP activation process can be separated into two steps: the TNAP protein is first stabilized by ZNT5-ZNT6 heterodimers or ZNT7 homodimers in the early secretory pathway and then is metallated by zinc supplied by both ZNT complexes [93] (Figure 4). In this two-step mechanism, the Pro-Pro (PP)-motif in luminal loop 2 of ZNT5 (which corresponds to luminal loop 7, because of the fact that ZNT5 has extra N-terminal TM helices [61]) and ZNT7, which is highly conserved in ZNT5 and ZNT7 homologs across multiple species, is thought to be important [100]. In model structures of ZNT5 and ZNT7, the PP-motif is located just above the intramembranous zinc-binding site in the TM helices [100], suggesting that a unique cooperative mechanism might operate between the intramembranous zinc-binding site and the PP-motif in the activation of TNAP. In contrast, a similar two-step activation mechanism does not seem to operate in the activation of ATX, because the ATX protein is not destabilized in cells lacking both ZNT5-ZNT6 heterodimers or ZNT7 homodimers, and so the PP-motif plays a somewhat minor role [24]. This discrepancy between TNAP and ATX may be explained by differences in the degree of their glycosylation, although this needs to be clarified in future studies.

*Znt5* or *Znt7* knockout (KO) mice show various phenotypes [101,102,103,104,105], but the association of those phenotypes with the early secretory pathway functions is unclear to date. One exception is the phenotype of osteopenia [101], which may be associated with reduced TNAP activity caused by a lack of ZNT5-ZNT6 heterodimers.

## 6. Importance of ZNT Transporters in Zinc-Related Regulated Secretory Pathway: After the Early Secretory Pathway

There are a number of cells that accumulate large amounts of zinc in cytoplasmic vesicles/granules (Figure 1). One can think that zinc, which is transported to the early secretory pathway, traffics those vesicles/granules through the secretory pathway and gets accumulated there. However, this is not the case. In fact, specific ZNT transporters are localized to the specific vesicles/granules and perform specific functions. In this section, representative vesicles/granules and the ZNT transporters involved in their function are briefly summarized to emphasize these points. Other aspects of this have been extensively reviewed elsewhere [1,10,11,12].

Insulin granules in pancreatic islet β-cells require high amounts of zinc in order to form insulin-zinc crystals, a process in which ZNT8 plays an indispensable role [106,107,108,109,110]. Nevertheless, a clear and crucial role for ZNT8 in regulating glucose homeostasis is yet to be established (*Znt8* KO mice are largely glucose tolerant), and thus the physiological relevance of zinc accumulation in insulin secretory granules remains unclear. Although, an interesting hypothesis is that zinc, which is secreted in concert with insulin, suppresses the insulin clearance in the liver by inhibiting clathrin-dependent insulin endocytosis [110]. Alterations in ZNT8 function are thought to lead to an increase in the risk of type 2 diabetes [111,112], because the R325W polymorphism in ZNT8 is associated with an increased risk of type 2 diabetes [113], and the R-form (i.e., the increased-risk form of ZNT8) likely alters its zinc transport activity [111,112]. In addition, another study has shown that haploinsufficiency of ZNT8 is protective against type 2 diabetes [114]. The relationship between ZNT8 and type 2 diabetes therefore requires further investigation [115]. The transporters ZNT5 and ZNT7 are also relatively highly expressed in pancreatic β-cells, [61,116,117], and both may be associated with β-cell function: loss-of-function of *Znt5* is associated with attenuation of the incidence of diabetes and mortality [103], whereas loss-of-function of *Znt7* impairs glucose tolerance and reduces glucose-stimulated increases in plasma insulin levels, hepatic glycogen levels, and pancreatic insulin content [104,105,118]. Moreover, loss-of-function of *Znt7* results in a markedly-reduced zinc content in β-cells, which is made more profound by the combined loss of function of *Znt8* [118]. ZNT5 and ZNT7 may therefore contribute to β-cell function in the early secretory pathway, but not in the insulin granules themselves.

Synaptic vesicles present in a subset of glutamatergic neurons in the hippocampus and neocortex also accumulate high amounts of zinc, which is mediated by ZNT3: *Znt3* KO mice lack synaptic zinc [119]. Zinc secreted from the synaptic vesicles into the extracellular space, as a result of ZNT3 activity, acts as a signaling molecule by modulating neuronal transmission and plasticity through binding to multiple ion channels, transporters, and receptors on postsynaptic neurons involved in neurotransmission [120,121,122]. The importance of synaptic zinc has been confirmed in knock-in mice studies, using glycine and *N*-methyl-d-aspartate receptors, in which the zinc-binding sites in each receptor were mutated [123,124]. A disturbance of synaptic zinc homeostasis or a dysfunction in ZNT3 has been suggested to result in neurodegenerative diseases [125,126,127].

Because zinc is essential for the growth and health of neonates, breast milk contains high amounts of zinc, considerably higher than the levels found in serum. The transporter ZNT2 is responsible for supplying zinc to the breast milk produced by the mammary epithelial cells in humans. Mothers with missense or nonsense mutations in the *ZNT2* gene secrete zinc-deficient milk (75–95% reduction), and thus infants exclusively breast-fed by mothers carrying the mutation experience transient neonatal zinc deficiency (TNZD; OMIM 608118) [87,128,129,130,131]. Zinc-deficient milk is produced by mothers with a heterozygous mutation in the *ZNT2* gene, and their infants also suffer from TNZD, suggesting that having one active copy of the *ZNT2* gene is not sufficient to provide zinc levels in breast milk adequate to support normal infant growth. One report has also suggested the involvement of ZNT5-ZNT6 heterodimers in the pathogenesis of TNZD [132], but there are no reports indicating that low-zinc breast milk can be attributed to mutations in the *ZNT4* gene in humans, although Znt4 is involved in low-zinc breast milk in mice [97,98].

Zinc that accumulated in granules/vesicles can be released in response to various stimuli and thereby regulate a number of diverse processes [110,133,134]. This phenomenon can be divided into two classes, the first being zinc secretion into the extracellular environment (e.g., zinc “sparks”) [133,134], as described for synaptic zinc, and the second being zinc release from intracellular stores into the cytosol (e.g., zinc “wave”) [135]. The latter phenomenon is strongly associated with the signaling functions of zinc and thus contributes to driving major signaling pathways [71,72,136]. The function of zinc in signaling has been extensively reviewed [2,41,137].

## 7. Perspectives

This review focuses on crucial functions of specific ZNT and ZIP zinc transporters in the early secretory pathway. As we have shown, these two classes of zinc transporter are doubtless key molecules required for the proper function of the early secretory pathway. However, there are many unsolved and fundamental questions that remain to be addressed. Specifically, how do both zinc deficiency and elevation in the early secretory pathway cause and exacerbate ER stress? How do those ZNT and ZIP transporters properly regulate zinc metabolism in a spatiotemporal manner in the early secretory pathway? Moreover, how is zinc coordinated in zinc-requiring ectoenzymes in the early secretory pathway? Are zinc chaperones required to facilitate zinc metallation of the large number of nascent proteins found in the early secretory pathway? Even the most fundamental question as to what the actual zinc concentration is in the early secretory pathway has not yet been definitively addressed, because the proposed zinc concentrations in the ER and the Golgi are controversial [138,139]. Moreover, clarification of a functional relationship(s) between the early secretory pathway and constitutive secretory or the regulated secretory pathway is required from the perspective of zinc metabolism. The answers to these questions can help our understanding of zinc in the early secretory pathway, and provide information that should be useful for the treatment of numerous diseases.

## Figures and Tables

**Table 1:** Relationship between properties of zinc-requiring enzymes and ZNT transporters *.

Enzyme	Active Site	Zn Coordination Residues	ZNTs Involved in Activation	Defects Caused by Loss of ZNTs
ALP	Bimetallic center	Asp, His, His for Zn_1_ His, Asp, Asp for Zn_2_	ZNT5-ZNT6, ZNT7	Loss of enzyme activity Protein destabilization
ATX CAIX	Bimetallic center Mononuclear Zn	Asp, His, His for Zn_1_, His, Asp, Asp for Zn_2_ His, His, His	ZNT5-ZNT6, ZNT7 ZNT4, ZNT5ZNT6,ZNT7	Loss of enzyme activity Decreases in enzyme activity
MMP-2 MMP-9	Mononuclear Zn **	His, His, His	ZNT5-ZNT6, ZNT7	Loss of enzyme activity Protein destabilization ***

* The structures of the zinc-requiring ectoenzymes are predicted based on homologous proteins; ** MMP-2 and MMP-9 have an additional zinc for stability of protein structure; *** Zinc supplementation partially restores both enzyme activity and protein stability. Zn: zinc.

## Abbreviations

ALP	Alkaline phosphatase
ATX	Autotaxin
CAIX	Carbonic anhydrase IX
CDF	Cation diffusion facilitator
ER	Endoplasmic reticulum
KO	Knockout
MMP	Matrix metalloproteinase
PP	Pro-Pro
S(E)R	Sarco(endo)plasmic reticulum
SLC	Solute carrier
TM	Transmembrane
TNAP	Tissue non-specific alkaline phosphatase
TNZD	Transient neonatal zinc deficiency
UPR	Unfolded protein response
ZIP	Zrt- and Irt-like protein
ZNT	Zn transporter
